# Loss of job-related right to healthcare associated with employment turnover: challenges for the Mexican health system

**DOI:** 10.1186/s12913-018-3283-7

**Published:** 2018-06-15

**Authors:** Germán Guerra, Emilio Gutiérrez-Calderón, Nelly Salgado de Snyder, Víctor Hugo Borja-Aburto, Adolfo Martínez-Valle, Miguel Ángel González-Block

**Affiliations:** 1 National Institute of Public Health, Centre for Health Systems Research, Av. Universidad 655. Col. Santa María Ahuacatitlán, CP 62100 Cuernavaca, Morelos Mexico; 2 Independent consultant, Barranca del Muerto 231. Col. San José Insurgentes, CP 03900 Mexico City, Mexico; 3 Unidad de Atención Primaria a la Salud, Instituto Mexicano del Seguro Social, Hamburgo 18. Col. Juárez, Cuauhtémoc, CP 06600 Mexico City, Mexico; 4 Secretaría de Salud, Lieja 7, Colonia Juárez, CP 06600 Mexico City, Mexico; 5 Universidad Anahuac Mexico, Av. Universidad 46. Col. Lomas Anáhuac, CP 52760 Huixquilucan, Edomex Mexico

**Keywords:** Healthcare utilization, Chronic illness, Informal sector, Labour markets

## Abstract

**Background:**

The Mexican health system segments access and right to healthcare according to worker position in the labour market. In this contribution we analyse how access and continuity of healthcare gets interrupted by employment turnover in the labour market, including its formal and informal sectors, as experienced by affiliates to the Mexican Institute of Social Security (IMSS) at national level, and of workers with type 2 diabetes (T2DM) in Mexico City.

**Methods:**

Using data from the National Employment and Occupation Survey, 2014, and from IMSS electronic medical records for workers in Mexico City, we estimated annual employment turnover rates to measure the loss of healthcare access due to labour market dynamics. We fitted a binary logistic regression model to analyse the association between sociodemographic variables and employment turnover. Lastly we analysed job-related access to health care in relation to employment turnover events.

**Results:**

At national level, 38.3% of IMSS affiliates experienced employment turnover at least once, thus losing the right to access to healthcare. The turnover rate for T2DM patients was 22.5%. Employment turnover was more frequent at ages 20–39 (38.6% national level; 28% T2DM) and among the elderly (62.4% national level; 26% T2DM). At the national level, higher educational levels (upper-middle, OR = 0.761; upper, OR = 0.835) and income (5 minimum wages or more, OR = 0.726) were associated with lower turnover. Being single and younger were associated with higher turnover (OR = 1.413). T2DM patients aged 40–59 (OR = 0.655) and with 5 minimum wages or more (OR = 0.401) experienced less turnover. Being a T2DM male patient increased the risk of experiencing turnover (OR = 1.166). Up to 89% of workers losing IMSS affiliation and moving on to other jobs failed to gain job-related access to health services. Only 9% gained access to the federal workers social security institute (ISSSTE).

**Conclusions:**

Turnover across labour market sectors is frequently experienced by the workforce in Mexico, worsening among the elderly and the young, and affecting patients with chronic diseases. This situation needs to be prospectively addressed by health system policies that aim to expand the financial health protection during an employment turnover event.

## Background

According to the Commission on Social Determinants of Health of the World Health Organization, labour markets and their policies, along with the working and employment conditions, have a considerable impact on health and health equity [1]. When healthcare financing depends on social security arrangements, the affiliation, access and utilisation of healthcare services by the population is determined mostly on the terms of employment [2].

A trait shared by low and middle income countries is the segmentation of the workforce into formal and informal labour markets [3]. The formal market is characterised by access to contributory or tax-based social protection schemes (including insurance for healthcare, disability, life, workers’ compensation plans, day-care and pensions) with varying degrees of coverage, gained through the type of work or employment activities performed in public or private institutions and business, and reported to the taxing authority [4]. The informal labour market is defined by the exclusion of access to such schemes and its reliance on out-of-pocket expenditure to access private services as well as on government support provided through social assistance programmes such as safety nets, cash transfers and voluntary contributory schemes to facilitate access to packages of limited health services. This segmentation has been considered a problem in Mexico because it excludes large population groups from social security rights and restricts access to government-subsidised healthcare, in a context where turnover across formal and informal labour market segments can occur [5]. Arguably, participating in the formal or informal labour markets can be considered a social determinant of health since access to health services and related health outcomes depend on the conditions of employment [6–9].

The Mexican government defines the workforce in the informal labour market as the proportion of the population that either is considered vulnerable because of the working and employment conditions of their workplace, or is not recognised by employers or their source of work as being under an employment relationship [10]. According to this definition, 62.3% of the total employed population in Mexico participates in the informal sector [11].

The high proportion of informality in the Mexican labour market occurs as a result of the asymmetry in social rights across the workforce. The Mexican Constitution of 1917 enshrined healthcare as a shared responsibility of employers and employees following the model legislated in Germany under Bismarck in 1883. In 1929, the Constitution was modified to spur economic growth through the federalising of labour legislation and mandating a federal social security law [12]. This law was enacted in 1943, creating the Mexican Institute of Social Security (IMSS) and leading to the integration of all social security schemes, except those of the most powerful industries such as banks, railroad, oil, electricity and beer companies, which retained their own schemes. Informal sector workers were offered limited government assistance through the Ministry of Health and Social Assistance. In 1959, federal worker social security schemes were integrated in a separate national institution, the Instituto de Seguridad y Servicios Sociales de los Trabajadores del Estado (known as ISSSTE), while local (state and municipal) governments developed arrangements of their own to protect their employees. In 1983, Article 4 of the Constitution was amended to include the right to health protection for all Mexicans, thus empowering a new Ministry of Health to coordinate the health sector as a whole and to increase the range of non-contributory services as well as voluntary affiliation to alternative health financial protection schemes.

It is important to note that, in Mexico, labour market and social security segmentation go hand-in-hand because social security institutions and government assistance programmes are vertically integrated and provide access to exclusive medical and social services. This policy model assumes that most of the workforce remains static within each social security or social assistance scheme along significant periods of time, and that beneficiaries seek healthcare mostly through providers corresponding to the institutions related to their labour market segment. However, Levy-Algazy [5] reported that, within a one-year period between 2005 and 2006, up to 20% of IMSS workers moved from the formal labour market segment to informality or to economic inactivity, thus losing access to IMSS healthcare. Furthermore, insured workers often utilise private healthcare providers or Ministry of Health facilities, leading to service duplication, high out-of-pocket expenditure and decreased incentives to improve insured services [13–15].

Persistently high levels of informality may be tied to high levels of employment turnover between formal and informal sectors. In turn, precarious working conditions in the informal sectors might lead to workforce turnover towards economic inactivity, with the corresponding loss of social benefits and continuity of care. This situation may be particularly problematic for Mexico, a country where chronic diseases account for the highest share of mortality and disability [16]. Previous studies [17, 18] have highlighted the sociodemographic factors associated with employment turnover towards unemployment in Mexico, indicating that a larger proportion of women experience unemployment more frequently when compared to men, while continuous employment trajectories are associated to higher income, education and age, as well as to being married. Work just published suggest that employment turnover by IMSS-affiliated workers (IAW) in Mexico City is associated to a 43% decrease in the quality of care received by patients with diabetes and with a 19% decrease in diabetes control [19].

The present article analyses the frequency and social determinants of employment turnover in the Mexican labour market, including its formal and the informal sectors, in a cohort of IMSS-affiliated workers (IAW) across the calendar year 2014. Employment turnover allow us to approach the extent to which, the loss and the subsequent gain of healthcare rights following changes in employment status, is experienced within the Mexican workforce. Access to health services other than IMSS is analysed in relation to turnover events. We focus on estimating the frequency and determinants of employment turnover of IAW from affiliation to disaffiliation at the national level and particularly by IMSS affiliates diagnosed with type 2 diabetes mellitus (T2DM) in Mexico City. T2DM is Mexico’s first cause of disability adjusted life years as well as the second cause of mortality [16].

## Methods

### Employment turnover

Based on works by Short, Robertson, and the Organisation for Economic Co-operation and Development [20–22], we define employment turnover as the transition event from being employed in the formal labour market segment, to another employment status, including working in the informal labour market, over a particular period. When quantification is performed at the end of the observed period (t_+ 1_), in relation to an initial stock of persons at the beginning of the observed period (t_0_), a turnover rate can be calculated. Sources of information on IMSS affiliation status allows for the estimation of employment turnover among the IAW, and also of the change of access to healthcare other than IMSS. We approach formal/informal turnover by measuring the annual employment turnover rate (AETR) of the IAW, which is defined as follows:

$$ \mathrm{AETR}=\frac{\sum {\mathrm{IAW}}_{\mathrm{t}+1}\mid \mathrm{T}\ge 1}{\sum {\mathrm{IAW}}_{\mathrm{t}0}\mid \mathrm{T}=0}\times 100 $$

Where:

IAW_t0_ is any IAW with access to IMSS health services as a current job benefit at the beginning of the observed period;

IAW_t + 1_is any IAW who experienced employment turnover at least once during the observed period;

T is the number of times an individual experienced turnover between “t_0_” and “t + 1”;

t_0_ is the beginning of the observation period and;

t_+ 1_ is the end of observation period.

The intensity of the employment turnover is also measured by counting the times an individual experiences the loss of access to IMSS healthcare services in the period under study. The measurement of turnover intensity indicates the distribution of the initial stock of the IAW according to the frequency of turnover events. The annual employment turnover intensity rate (AETIR_n_) is defined as the percentage of the IAW that experience an event of employment turnover “n” times, between “t_0_” and “t + 1” as follows:

$$ {\mathrm{AETIR}}_{\mathrm{n}}=\frac{\sum {\mathrm{IAW}}_{\mathrm{t}+1}\mid \mathrm{T}=\mathrm{n}}{\sum {\mathrm{IAW}}_{\mathrm{t}0}\mid \mathrm{T}=0}\times 100 $$

Where IAW_t + 1_ is any IAW individual that experienced employment turnover “n” times during the observation period, for n = {1,2,3,4}.

### Longitudinal data

The calculation of the AETR and AETIR_n_ uses longitudinal data that registers the frequency of turnover events along time. We used data from the National Survey of Occupation and Employment (ENOE) to analyse employment turnover among the IAW at the national level. The ENOE is a trimonthly survey applied to a representative household sample in Mexico with the objective of obtaining statistical information on the occupational characteristics and determinants of the population at the national level [23].

The period of observation we chose was from the last trimester of 2013 (“t_0_”) to the last trimester of 2014 (“t_+ 1_”). We selected a subset of individuals (as described below) and obtained a longitudinal subsample to calculate the AETR and conduct statistical analysis.^1^ The inclusion criteria for the subsample were (1) any IAW aged 15 or older at time “t_0_”, (2) who declared having access to healthcare through their current job at time “t_0_”, and (3) who completed the survey across the five visits between t_0_ and t_+ 1_. IAW were excluded from the subsample if they did not meet these criteria. Our final subsample was an IAW cohort of 8347 individuals that had access to IMSS healthcare services at time “t_0_”.

It is important to note that, for each trimester, ENOE asks respondents to report their employment status within the previous week. This might result in an underestimation of turnover intensity, as the individual could have experienced several turnover cycles within the three-month period. Thus, turnover rates may actually be higher than observed. Given that no information is provided about access to healthcare in the last three months, we assume that the turnover entails no healthcare access during the period.

Employment turnover for IMSS patients diagnosed with T2DM was obtained from the IMSS affiliation department database containing dates of affiliation and disaffiliation across year 2014, for 11,045 workers with T2DM identified through electronic medical records in six IMSS Family Medicine Clinics (FMCs) in Mexico City. The sample frame to identify the six clinics consisted of the 53 FMCs in Mexico City with 10 or more examining rooms (23 FMCs in the south and 20 FMCs in the north of the city). The FMCs were categorised according to geographic location (south and north) and the number of examining rooms (10–19, 20–29, and ≥ 30 examining rooms). One FMC was chosen from each category through simple random selection. The affiliation data for all workers with a T2DM diagnosis was requested to IMSS using their affiliation number as key.

The inclusion criteria for this analysis were (1) having healthcare access to IMSS as an insured worker and (2) being a T2DM patient. The exclusion criteria were (1) having healthcare access to IMSS with less or equal to 15 days of insurance, (2) having IMSS student insurance, (3) having a voluntary family IMSS insurance, (4) being an IMSS pensioner or IMSS pensioner beneficiary, or (5) being a beneficiary of an insured worker.

### Statistical analysis

Our analysis of both ENOE and electronic medical records included a description of the IAW annual turnover and turnover intensity, analysing the associations with sociodemographic characteristics. We calculated the general AETR and AETIR_n_ by sex, age groups, daily minimum wage, and educational level. We analysed the sociodemographic factors associated with employment turnover by fitting a binary logistic regression model. Employment turnover as a dependent dichotomous variable (individual experiences turnover vs. does not experience turnover) was analysed with the variables of sex, highest educational level attained, marital status, age group, and daily income in minimum wages. Education and marital status were not available from the electronic medical records and were thus not included in this analysis.

Finally we described the access to other healthcare services for the workforce that lost IMSS affiliation due to employment turnover. We estimated the distribution of access to healthcare for the IAW that experienced employment turnover at the last trimester of 2014. If access was gained to another social security institution, the name of the institution was provided. The distribution includes also IAW that did not regain right to access to healthcare as employment benefit, or became economically not active or unemployed. The distribution was described according to the same sociodemographic characteristics used for AETR and AEITR_n_. We used IBM-SPSS statistical package version 22 for all mentioned analysis.

## Results

Employment turnover and employment turnover intensity (AETR and AETIR_n_) rates for the general IAW are shown in Table 1. The AETR was 38.3% in general and 39.2% for women and 37.8% for men. By age group, the employment turnover was higher among the youngest (15–19) and oldest age (60+) groups, at 65.4 and 62.4%, respectively.

**Table 1:** Annual employment turnover rate (AETR) and annual employment turnover intensity rate (AETIR_n_) by sociodemogaphic characteristics of IMSS-affiliated workers, Mexico, 2014 (*N* = 8347)

	Continuous affiliation to IMSS (Turnover events = 0)	Test of equality of proportions^a^	AETR (Percentage)	Test of equality of proportions^a^	AETIR_1_ (Percentage)	Test of equality of proportions^a^	AETIR_2_ (Percentage)	Test of equality of proportions^a^
General	61.7		38.3		33.0		5.3	
Sex								
*Male*	62.2	*a*	37.8	*a*	32.7	*a*	5.1	*a*
*Female*	60.8	*a*	39.2	*a*	33.7	*a*	5.5	*a*
Age group								
*15–19*	34.6	*a*	65.4	*a*	53.3	*a*	12.1	*a*
*20–39*	61.4	*b*	38.6	*b*	33.6	*b*	5.0	*b*
*40–59*	66.2	*c*	33.8	*c*	29.0	*c*	4.8	*b*
*60 or over*	37.6	*a*	62.4	*a*	52.8	*a*	9.6	*a*
Daily minimum wage^b^								
*Less or equal than 3*	59.0	*a*	41.0	*a*	35.4	*a*	5.6	*a*
*Greater than 3 and up to 5*	64.8	*b*	35.2	*b*	30.6	*b*	4.6	*a*
*Greater than 5*	68.7	*b*	31.3	*b*	26.1	*c*	5.2	*a*
Highest educational level attained								
*Incomplete elementary*	51.4	*a*	48.6	*a*	40.1	*a*	8.5	*a*
*Completed elementary*	57.7	*a,c*	42.3	*a*	35.6	*a*	6.7	*a,b*
*Completed secondary*^c^	64.5	*b*	35.5	*b*	30.9	*b*	4.6	*b*
*Completed upper-middle*^d^ *and upper*	61.3	*c*	38.7	*a,b*	33.6	*a,b*	5.1	*a,b*

Source: Own calculations based on National Survey of Occupation and Employment (ENOE). Trimesters: IV 2013 to IV 2014

^a^Values for the same variable and column not sharing the same subscript are significantly different at *P* < 0.05 in the two-sided test of equality for proportions

^b^Minimum wage in 2013: $64.76 MXN (US$3.37, exchange rate 2017). Minimum wage in 2014: $67.29 MXN (US$3.50, exchange rate, third trimester, 2017)(Servicio de Administración Tributaria, 2017)

^c^Equivalent to 9 years of formal schooling

^d^Equivalent to 12 years of formal schooling

A total of 33% of the IAW at national level experienced employment turnover once, and only 5.3% experienced it at least twice during the observed period. Employment turnover was much more intense for the 60 or over age group (AETIR_1_ = 52.8%). The 15–19 age group experienced similar levels of turnover as the oldest group, as 53.3% of the youngest IAW reported having at least one employment turnover event during the calendar year 2014, and 12.1% experienced it at least twice. T2DM patients followed this age-related turnover pattern (Table 2), but employment turnover was experienced less often, not reaching 30% in any age group (28% for ages 20–39, and 26% for 60 or over).

**Table 2:** Annual employment turnover intensity rate by sociodemogaphic characteristics of T2DM patients at FMC, Mexico City, 2014 (*N* = 11,045)

	Continuous affiliation to IMSS(Turnover events = 0)	Test of equality of proportions^a^	AETIR_1_^b^(Percentage)	Test of equality of proportions^a^
General	77.5		22.5	
Sex				
*Male*	77.1	*a*	22.9	*a*
*Female*	78.0	*a*	22.0	*a*
Age group				
*20–39*	72.0	*a*	28.0	*a*
*40–59*	79.5	*b*	20.5	*b*
*60 or over*	74.0	*a*	26.0	*a*
Daily minimum wage^c^				
*Less or equal than 3*	72.5	*a*	27.5	*a*
*Greater than 3 and up to 5*	78.7	*b*	21.3	*b*
*Greater than 5*	86.5	*c*	13.5	*c*

Source: Own calculations based on IMSS electronic medical records, 2014

^a^Values for the same variable and column not sharing the same subscript are significantly different at *P* < 0.05 in the two-sided test of equality for proportions

^b^Note that only AETR was calculated for T2DM patients, thus AETR = AETIR_1_

^c^Minimum wage in 2013: $64.76 MXN (US$3.37, exchange rate 2017). Minimum wage in 2014: $67.29 MXN (US$3.50, exchange rate, third trimester, 2017) (Servicio de Administración Tributaria, 2017)

Employment turnover intensity and daily minimum wage levels among IAW showed an inverse proportional relation as the employment turnover decreased with the number of daily minimum wages earned. However, it is worth noting that differences between categories of number of minimum wages became statistically significant only after earning more than three minimum wages. T2DM patients followed the same relation, but employment turnover was also experienced less often (Table 2). Finally, educational level (Table 1) showed a similar pattern to income, except for the upper-middle and upper levels, where there was a slightly higher employment turnover than complete secondary level for all intensity levels.

### Sociodemographic factors associated to employment turnover

Table 3 shows the sociodemographic factors associated to employment turnover of IAW. The characteristics associated with less risk of employment turnover were having higher schooling grade (upper-middle, OR = 0.761; upper, OR = 0.835) and earning 3–5 daily minimum wages (OR = 0.852) or higher than 5 minimum wages (OR = 0.726). Being single and younger were associated with higher risk of employment turnover (single, OR = 1.413).

**Table 3:** Sociodemogaphic factors associated to employment turnover of IMSS-affiliated workers, Mexico, 2014 (*N* = 8347)

Sociodemographic factor^a^	Category	Odds ratio
Age group	*15–19*	Reference category
	*20–39*	0.399***
	*40–59*	0.347***
	*60 or over*	1.032
Daily minimum wage	*Less or equal than 3*	Reference category
	*Greater than 3 and up to 5*	0.852**
	*Greater than 5*	0.726***
Highest schooling grade attained	*Elementary*	Reference category
	*Upper-middle*	0.761***
	*Upper*	0.835*
Marital status	*Married or consensual union*	Reference category
	*Divorced or separated*	1.013
	*Widowed*	0.830
	*Single*	1.413***

Source: Own calculations based on National Survey of Occupation and Employment (ENOE). Trimesters: IV 2013 to IV 2014

^a^Sex was not statistically significant (*P* ≥ 0.05)

*P < 0.05

**P < 0.01

***P < 0.001

The sociodemographic factors associated to employment turnover of T2DM patients are shown in Table 4. Being in the 40–59 age group (OR = 0.655) and having a higher income than three or more daily minimum wages were associated with less risk of turnover; (3–5 daily minimum wages, OR = 0.691; higher than 5 minimum wages, OR = 0.401). Male T2DM patients experienced a higher risk of employment turnover than female T2DM patients (OR = 1.166).

**Table 4:** Sociodemogaphic factors associated to employment turnover of T2DM patients at FMC, Mexico, 2014 (*N* = 11,045)

Sociodemographic factor	Category	Odds ratio
Sex	*Female*	Reference category
	*Male*	1.166***
Age group	*20–39*	Reference category
	*40–59*	0.655***
	*60 or over*	0.818*
Daily minimum wage	*Less or equal than 3*	Reference category
	*Greater than 3 and up to 5*	0.691***
	*Greater than 5*	0.401***

Source: Own calculations based on IMSS electronic medical records, 2014

*P < 0.05

***P < 0.001

### Access to job-related healthcare services associated to turnover events

Out of the total turnover events reported in the last semester, 61.7% led to another job, however almost 89% of them failed to access job-related health services (Table 5). The ISSSTE was the most frequent source of job-related health services. More men than women remain employed (71.3% vs.46.6%). However, when employed, a higher proportion of women gained job-related healthcare services than men (16.4% vs. 9.3%). It is worth noting that 13.8% of women acquired healthcare services by ISSSTE.

**Table 5:** Access to job-related healthcare services of IMSS-affiliated workers that experienced turnover events, Mexico, last trimester, 2014. Percentage frequency distribution, (*N* = 1913)

	Employment situation		Access to job-related health services among the employed		
	Employed	Unemployed or not economically active	ISSSTE	Another social security institution	Without job-related access*
General	61.7	38.3	9.1	2.3	88.6
Sex					
*Male*	71.3	28.7	7.1	2.2	90.8
*Female*	46.6	53.4	13.8	2.6	83.6
Age group					
*15–19*	47.7	52.3	0.0	0.0	100.0
*20–39*	60.9	39.1	9.3	2.6	88.1
*40–59*	68.0	32.0	10.3	2.3	87.4
*60 or over*	45.8	54.2	4.5	0.0	95.5
Daily minimum wage^a^					
*Less or equal than 3*	58.9	41.1	5.6	2.0	92.4
*Greater than 3 and up to 5*	66.7	33.3	10.8	1.7	87.5
*Greater than 5*	62.7	37.3	11.6	5.0	83.5
Highest educational level attained					
*Completed elementary*	67.6	32.4	2.9	1.0	96.2
*Completed secondary or upper-middle*^b^	60.7	39.3	6.4	2.1	91.5
*Upper or more*	60.2	39.8	18.9	3.5	77.6

Source: Own calculations based on National Survey of Occupation and Employment (ENOE). Trimesters: IV 2013 to IV 2014

^a^Minimum wage in 2013: $64.76 MXN (US$3.37, exchange rate 2017). Minimum wage in 2014: $67.29 MXN (US$3.50, exchange rate, third trimester, 2017)(Servicio de Administración Tributaria, 2017)

^b^Equivalent to 12 years of formal schooling

*Difference in extreme value proportions are statistically significant at *p* < 0.05

Age makes an important difference in the outcome of turnover events. Among workers between 15 and 19 years of age, 47.7% remain employed as against 68% of those 40 to 59 years of age. Up to 87.4% of workers 40 to 59 years of age that remained employed failed to gain access to job-related health services, and all of the youngest lost this type of access. Regarding income, lowest earners fail to gain job-related health service access in a proportion as high as 92.4% against 83.5% among the lowest earners. Education levels and continuous employment show small differences between categories, but this sociodemographic factor is the largest differentiator with respect to job-related health service access. For instance the gap in access to ISSSTE between the least and the most educated is 16%.

## Discussion

To our knowledge, this is the first study to analyse employment turnover across the formal and informal labour markets as defined by social security affiliation, as well as the loss of access to healthcare services within vertically integrated health systems and the labour market dynamics affecting it. The study was able to utilise large samples from both the ENOE and IMSS medical records, obtaining reliable estimates.

With a general annual turnover rate of 38.3 per 100 affiliated workers, social security seems to be failing to fully protect its beneficiaries on a continuous basis. Furthermore, 88.6% of turnover events lead to informal jobs that fail to provide access to health services. This situation is of much greater concern among those aged 60 and above, for whom AETR is as high as 62.4% and where 54.2% leave the economically active population or become unemployed. Among the population in the oldest age group that remain employed, 95.5% fail to gain access to job-related health services.

Employment turnover among workers with T2DM in Mexico City was 22.5%, a rate 41% lower with respect to general workers nationwide. This result can be interpreted as a strategy by workers with greater healthcare needs to retain their source of medical care. However, this assumption has to be carefully interpreted as the labour market in Mexico City may be more stable, away from seasonal agro-industrial work undertaken elsewhere.

A 22.5% yearly turnover among T2DM patients is of particular concern, as this may reflect a loss of continuity of care for diabetes-control among workers exposed to relatively frequent disaffiliation. Additionally, the fact that elderly workers are much more prone to employment turnover suggests an even greater vulnerability [24], both in the general population and among those with T2DM. Further research needs to be undertaken across more comparable populations to ascertain the extent to which chronic diseases induce job stability, or if they hinder the capacity to maintain employment in the formal labour market.

We now have robust evidence demonstrating an association between employment turnover and chronic disease care in Mexico. Members or our team led by Doubova undertook a three-year follow-up study of 27,217 IMSS patients with a diagnosis of diabetes in Mexico City, assessing the relationship between quality of diabetes care processes and control [19]. The mean duration of disaffiliation to IMSS in the study period was of 120 days, not including the two-month grace period that IMSS provides to patients that lose affiliation. Study results show that patients experiencing employment turnover decreased their quality of diabetes care by 43.2%, measured as patients receiving less than 50% of standard disease control interventions. While such interventions could have been provided by alternative health providers while the patient was disaffiliated to IMSS, employment turnover had –in itself– a measurable and large impact on diabetes control, as evidenced by the fact that it was associated with a 19.2% reduction in the attainment of a standard set of physiological indicators.

Our estimates of employment turnover, specifically the AETR, are higher than those estimated by Levy-Algazi [5] for 2005–2006 (approximately 20%), likely due to methodological differences. Levy-Algazi reported data for only a four trimesters period, thus missing the situation of affiliation at the beginning of the period. Our study assessed affiliation from the last trimester of 2013 which is the initial period where the five visits across year 2014 started, thus covering a complete year of panel data available. The difference may also be due to a major stability in the labour market during the period studied by Levy-Algazi. Furthermore, our findings are different to those of previous studies assessing the stability of employment trajectories, which indicated that these were more stable for older populations [17, 18].

The sociodemographic factors associated to employment turnover are related to the so called “demographic dividend” stemming from a predominantly young population in Mexico, as well as to a phenomenon of a rapidly ageing population [25]. Indeed, employment turnover is more frequently experienced in younger age groups, but resurges among older age groups close to retirement age. Younger groups, specifically those aged 15–19, might be experiencing a more frequent turnover due to the type of work in which they engage (part-time) or due to competing interests in their life course such as pursuing further education. It could also be related to early fertility events, as Mexico has experienced a rise of the adolescent fertility rate in the period 2009–2014 of 7.84 births per 1000 women aged 15–19 (69.2 vs. 77.04) [26].

The loss of IMSS coverage at young ages is critical in the support of reproductive healthcare, among other needs. With regards to chronic diseases in general, the establishment of a trusted, stable relationship with a healthcare provider is critical for their early detection, diagnosis and effective control [24]. For older age groups, periodic social security disaffiliation entails greater costs to formal labour market re-entry and access to healthcare, as well as entitlement to a pension scheme [27].

In order to address employment turnover, innovative labour market policies are urgently required to absorb the growth in the Mexican working-age population. According to García [28], the working-age population in Mexico will reach a maximum of 60 million by 2050, with a subsequent steady decline in the decades to follow. For the period of 2007–2015 an average of 9.5 million jobs should have been created to absorb the Mexican demographic dividend of working-age cohorts. Yet, recent labour market policies have been unable to reach this goal. During the period 2007–2012 the net growth of formal labour market jobs registered by IMSS and the National Institute of Statistics and Geography (INEGI) was 1.6 million, with 32% being temporary jobs. For the first semester period of January 2012 to the first semester period of June of 2017, 2.1 million formal labour market jobs were created, with 17% being temporary [29]. The current Mexican president’s administration (Enrique Peña-Nieto) claims to have created 2 million additional jobs between 2013 and 2016, a figure higher than previous administrations [30], yet still far below national needs.

Several policies have been implemented in the last ten years to support the formalisation of the labour market. During Calderon’s administration, the “First Employment Program” was implemented between 2006 and 2012, subsidising social security contributions for first-time employees. Yet, the uptake of the programme was low, with only 83,000 workers registered in its first 3 years [31] and the programme has ceased to exist. The Peña Nieto administration is currently implementing a programme to promote the formalisation of small businesses through the Fiscal Incorporation Programme (*Régimen de incorporación fiscal*; RIF), including full healthcare and pension coverage for both employers and employees with the incentive of a 10-year period of federal fiscal and IMSS affiliation subsidies. In parallel, however, labour legislation enacted in November 2012 enabled greater contractual flexibility through outsourcing, seasonal employment, hourly wage rates and traineeships, thus challenging labour stability and the continuity of employment [32].

Policies to bolster labour market formalisation have thus far failed to meet the expectations to promote the growth of needed formal sector jobs, while policies favouring labour flexibility may in fact be working against formalisation as currently defined. Furthermore, these policies ignore the high permeability across the formal and informal labour markets, with the loss of access to social security benefits. Small firms perceive RIF as a barrier to formalisation and, along with opposition parties, are seeking legislation to promote formalisation based exclusively on income tax and fiscal simplification, possibly excluding subsidies for social security benefits. Recent RIF reforms enabled the deduction of private medical expenditures in tax declarations, which could signal an alternative to IMSS subsidies [33]. Proposals are also emerging to substitute RIF with increased value-added taxation in order to increase the financing of social services for informal sector workers and their families [34].

### Study limitations

Most of the limitations of the study stem from the specialised nature of each source of information. As the focus of ENOE is on the labour market, it was not possible to assess the actual use of IMSS healthcare services by respondents, but only their access. In the same way, ENOE does not specifically register if IAW keep access to healthcare as part of their previous job under the eight-week grace period, but it is relevant to know how this benefit is acted upon by IAW in order to provide more evidence about its adequacy to the labour market dynamics. Furthermore, workers could have lost access to IMSS directly, but could have retained it if they remained as a dependent of an IAW as the spouse or a child.

Although ENOE panel data allowed for a refined approach to track employment trajectories of IAW to inactivity, informality or to public employment, we focused solely on the loss of access to IMSS services and the immediate access to other job-related health services. Such trajectories need to be further investigated to have a more complete picture of the impact of employment turnover on job-related healthcare access.

Regarding T2DM patients estimates from IMSS electronic medical records, one limitation lies in the difficulty of measuring employment turnover of T2DM patients and making it comparable to the general IAW. The aim of the study was to assess the level of turnover within each population (National level and Mexico City), rather than comparing the two samples. Further research is required to analyse the role of chronic diseases, such as T2DM, as a cause or a consequence of employment turnover.

Another important limitation of this study is the lack of qualitative data to ascertain the extent to which employment turnover is associated to actual health service utilization, and to the perception of the quality of care, particularly in comparison to services provide by IMSS. This limitation is of concern given that a small but significant proportion of turnover events is associated to accessing ISSSTE services, while private and public assistance health services are widely available and could well offer an alternative to care for the majority of continuously employed workers that fail to access job-related health services.

Finally, as the majority of turnover events led to informal jobs without access to healthcare services, it is important to conduct further research in order to obtain more evidence in the context of our findings that points to a large impact of employment turnover on health indicators.

## Conclusions

Turnover from employment providing affiliation to IMSS to unemployment without direct IMSS affiliation is a frequent event in the Mexican labour market as attested by IMSS national level data, the largest formal sector insurer. Furthermore, the majority of turnover events are associated to the informal labour market and to the loss of job-related health care. High turnover rates reduce the effectiveness of social security, leading to frequent loss of access to benefits such as healthcare and to a reduced impact of health interventions. The most vulnerable population groups to employment turnover of IAW are the youngest and the oldest, the less educated, and those with the lowest income. Yet, these are precisely the groups that should be fully covered by social protection schemes.

Thus, employment turnover as a determinant of health is a consequence of the labour market dynamics, affecting continuity of access to health services in Mexico and challenging the quality of care. It is questionable whether reforms to labour market regulations as currently formulated and implemented can contribute to extending continuous access to trusted health providers. Alternatives must be sought to ensure universal access to essential, person-centred primary healthcare services, regardless of the source of funding or the position of individuals within the labour market. IAW losing this source of funding could retain access to IMSS primary care providers funded by Ministry of Health assistance programmes such as *Seguro Popular*, which already funds services at similar levels of healthcare on a per-affiliate basis.

Other options could include extending the right of access to healthcare beyond the current eight week period after loss of affiliation to social security institutions, thus enabling workers, and particularly those with chronic diseases or the elderly, to maintain continuity of care within IMSS, regardless of employment situation. Funding for such a scheme could be provided within IMSS through the permanent disability insurance fund, thus avoiding the disability that could be incurred from the documented loss of quality of care stemming from employment turnover.

## Abbreviations

AETIR: Annual employment turnover intensity rate
AETR: Annual employment turnover rate
ENOE: National Survey of Occupation and Employment
FMCs: Family Medicine Clinics
IAW: IMSS-affiliated workers
IMSS: Mexican Institute of Social Security
INEGI: National Institute of Statistics and Geography
ISSSTE: Instituto de Seguridad y Servicios Sociales de los Trabajadores del Estado
RIF: Fiscal Incorporation Programme
T2DM: Type 2 diabetes mellitus

## Footnotes

1 The National Survey of Occupation and Employment (ENOE) question number “6d” from the basic questionnaire: As part of your current job, do you have access to medical care at? 1) IMSS; 2) Pemex, navy or army hospital/clinic; 3) Instituto de Seguridad y Servicios Sociales de los Trabajadores del Estado (ISSSTE); 4) State ISSSTE; 5) other; 6) no access; 9) Does not know. This question allowed us to select the cohort of all individuals that met the criteria, and answered “IMSS” (option 1).
